# Recent Progress in Organic Photodetectors and their Applications

**DOI:** 10.1002/advs.202002418

**Published:** 2020-11-19

**Authors:** Hao Ren, Jing‐De Chen, Yan‐Qing Li, Jian‐Xin Tang

**Affiliations:** ^1^ School of Physics and Electronics Science Ministry of Education Nanophotonics & Advanced Instrument Engineering Research Center East China Normal University Shanghai 200062 P. R. China; ^2^ Jiangsu Key Laboratory for Carbon‐Based Functional Materials & Devices Institute of Functional Nano & Soft Materials (FUNSOM) Soochow University Suzhou Jiangsu 215123 P. R. China

**Keywords:** detection, flexibility, integrated optoelectronics, organic photodetectors, photomultiplication effect

## Abstract

Organic photodetectors (OPDs) have attracted continuous attention due to their outstanding advantages, such as tunability of detecting wavelength, low‐cost manufacturing, compatibility with lightweight and flexible devices, as well as ease of processing. Enormous efforts on performance improvement and application of OPDs have been devoted in the past decades. In this Review, recent advances in device architectures and operation mechanisms of phototransistor, photoconductor, and photodiode based OPDs are reviewed with a focus on the strategies aiming at performance improvement. The application of OPDs in spectrally selective detection, wearable devices, and integrated optoelectronics are also discussed. Furthermore, some future prospects on the research challenges and new opportunities of OPDs are covered.

## Graphical abstract

Recent progress in organic photodetectors is reviewed, including different device structures, features, and operation mechanisms. Benefiting from the improved performance, the applications of organic photodetectors for selective detection, wearability, and integrated devices are highlighted.

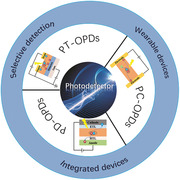

## 1 Introduction

Photodetectors show great significance in optoelectronic devices with the ability to convert light signals into electrical signal. They have diverse applications, such as optical communication, environmental monitoring, biomedical imaging, and sensing.^[^ ^1^, ^2^ ^]^ Traditional photodetectors exhibit excellent performance in terms of photosensitivity, responsivity, and detectivity by virtue of inorganic III‐V semiconductors that possess high carrier mobility, good stability, and small exciton binding energy.^[^ ^3^, ^4^, ^5^ ^]^ Moreover, the properties of inorganic photodetectors (IPDs) can be improved by designing suitable materials and introducing different physical mechanisms, such as piezo‐phototronic effect, avalanche effect, and impact ionization.^[^ ^6^, ^7^ ^]^ Besides, IPDs can integrate with complementary metal‐oxide semiconductors (CMOS) due to their great compatibility, which guarantees their application in miniaturized photodetectors and other featured devices.^[^ ^8^ ^]^ However, given the intrinsic drawbacks, e.g., high brittleness and complicated manufacturing process, IPDs are limited in flexible applications and cost‐saving.^[^ ^9^ ^]^ Besides, due to the broadband absorption of inorganic semiconductors, spectrally selective detection of IPDs is unavailable without attached optical filters.

To address the mentioned issues, photodetectors based on organic semiconductors are broadly investigated. The proposed organic photodetectors (OPDs) exhibit advantages of detection wavelength tunability, low‐cost manufacturing, and compatibility with lightweight and flexible devices. Ultraviolet (UV), visible, and near‐infrared (NIR) OPDs can be obtained by optimizing optical bandgap of organic semiconductors and device architecture of OPDs rather than attaching optical filters. Such an outstanding feature favorates to simplify the fabrication process of spectrally selective photodetectors and thus to lower the manufacturing cost. The mechanical flexibility of organic semiconductors benefits the realization of wearable photodetectors that has great application potential in health detection sensors. However, the low carrier mobility and the disordered molecular alignment of organic molecules lead to the relatively slower response speed and less charge generation in OPDs as compared to IPDs.^[^ ^10^, ^11^ ^]^ Accordingly, numerous efforts have been devoted to improving the properties of OPDs. Particularly, the attachment of a preamplifier circuit to OPDs is an efficient way to enhance the photosensitivity and detection.^[^ ^12^, ^13^ ^]^

**Figure 1:**
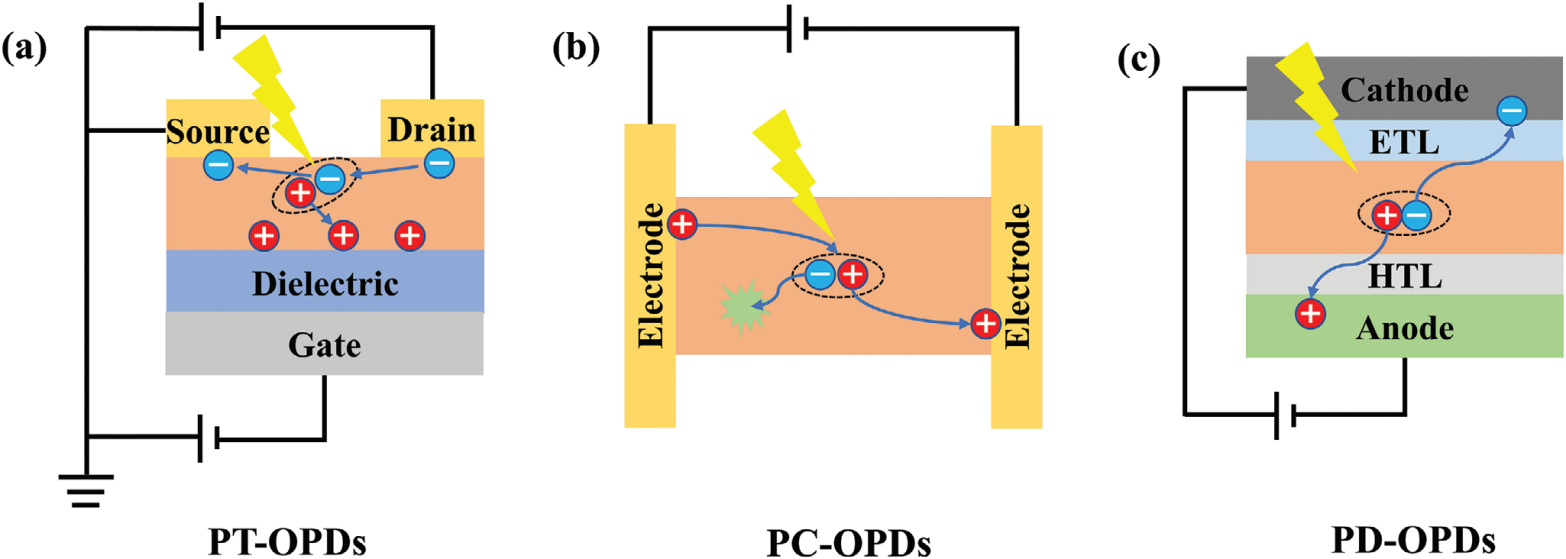
Schematic working mechanisms of a) PT‐OPDs, b) PC‐OPDs, and c) PD‐OPDs.

Various specific strategies have been investigated for the performance improvement of OPDs with different structures. As schematically shown in **Figure** **1**, OPDs can be classified into phototransistors‐based OPDs (PT‐OPDs), photoconductors‐based OPDs (PC‐OPDs), and photodiodes‐based OPDs (PD‐OPDs) with different working mechanisms and architectures that can meet different requirements of a variety of applications. From the perspective of the device structure, the majority of PT‐OPDs show three electrodes which are gate, source, and drain electrode. Notably, recent research realized phototransistor‐type perovskite photodiodes by using a two‐terminal architecture, which may inspire the device structure evolution of OPDs.^[^ ^14^ ^]^ In contrast, PC‐OPDs and PD‐OPDs are based on two electrodes (i.e., anode and cathode). Each kind of OPDs has its limitation and specialty in optical detecting. For PT‐OPDs, an additional photoconductive gain can be realized by using the three‐terminal structure and applying an additional electrical bias. Further introduced hybrid‐layered structures can facilitate charge separation and transportation, thereby improving the device performance. For PC‐OPDs, through trapping minority carriers and recycling maximum carriers, a photoconductive gain can also be achieved by photomultiplication (PM) effect that is considered as an efficient way to enhance the responsibility and detectivity of OPDs. Unlike IPDs, PM phenomenon cannot be achieved by the principle of avalanche effect and impact ionization in organic materials due to their disorder nature and high exciton binding energy.^[^ ^15^, ^16^ ^]^ While the existence of interfacial trap‐assisted charge tunneling injection could give rise to PM effect, resulting in high external quantum efficiency (EQE) over 100%. However, photoconductors have limited use in high‐frequency optical demodulators, such as in optical mixing. For low‐level detection at microwave frequencies, PC‐OPDs show a lower speed and signal‐to‐noise ratio. As the most widely researched OPDs, PD‐OPDs exhibit an extraordinary property in photosensitivity and photoresponsivity. But their EQE is theoretically less than 100% because of inevitable energy losses in the photoelectric conversion process.^[^ ^10^, ^17^ ^]^ Nevertheless, by rational designing device structure and neglecting the decrease of detectivity, PM effect also can be introduced to PD‐OPDs, breaking the EQE limitation. However, the response time is simultaneously increased due to the long‐lasting charge accumulation process. In the past decade, researchers have presented many methods, such as materials synthesis, interfacial modification, and structure engineering, to further improve the advantages and make up for the shortcomings of various kinds of OPDs.

In this review, we first introduce the respective operation mechanism of various kinds of OPDs. Corresponding advances in performance improvement are discussed simultaneously. By utilizing the innovative device structures, manipulated PM effect, and various superior materials, OPDs have obtained remarkable EQE, photoresponsivity, detection, etc. Then, the researches focused on special applications of OPDs including spectrally selective detection, wearable devices, integrated multifunctional devices are reviewed, which indicates the broad application scenarios of OPDs. Finally, a summary and a prospect of development and application of OPDs are given.

## 2 PT‐OPDs

### 2.1 Operation Mechanism

As an important type of OPDs, phototransistors‐based organic photodetectors (PT‐OPDs) feature the three‐terminal configurations (i.e., gate, source, and drain electrodes) as same as organic field‐effect transistors (OFET). In such devices, the channel resistance between the source and drain electrodes can be adjusted by applying bias voltage at the gate electrode, that is, channel conductance can be controlled by a gate terminal. Moreover, the transporting channel can also be modulated by light absorption of organic semiconductor that generates carriers. Thus, PT‐OPDs can exhibit high photocurrent and high responsivity due to the internal photoconductive gain, which also leads to the amplification of photocurrent and EQE values of over 100%.^[^ ^18^, ^19^, ^20^, ^21^ ^]^

The channel materials should possess both high carrier mobility and efficient light absorption to obtain high photosensitivity of PT‐OPDs. By rational designing the molecular structures and modifying the morphology of functional films, significant progress has been made. For instance, Zhang et al. fabricated a layered photodetector based on graphene/MoS_2_ heterostructure, in which excitons are generated under light absorption and separated in the interfaces between graphene and MoS_2_ layer. With applied perpendicular electric field, the photodetector exhibited excellent performance with a photoresponsivity of 10^7^ A W^−1^ and a gain of 10^8^ at room temperature.^[^ ^22^ ^]^ To achieve highly efficient OPDs, different structures have been intensively studied. They are roughly categorized into a layered structure, blended structure, and composite structure.

### 2.2 Layered Structure

**Figure 2:**
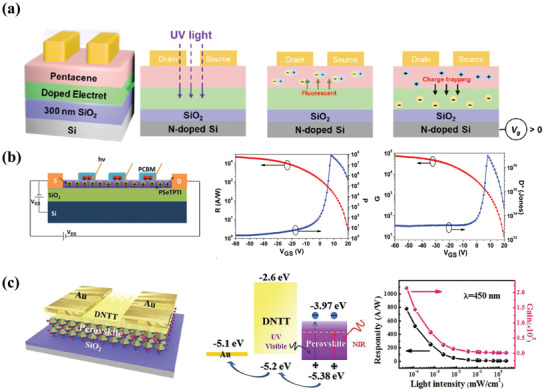
a) Schematic device structure and operation mechanism of PT‐OPDs. Reproduced with permission.^[^ ^26^ ^]^ Copyright 2019, The Royal Society of Chemistry. b) Schematic device structure, along with photoresponsivity and detectivity as a function of *V*_GS_. Reproduced with permission.^[^ ^27^ ^]^ Copyright 2015, Wiley‐VCH. c) Schematic device structure and operation mechanism of the hybrid PT‐OPDs, along with responsivity and gain as a function of incident light intensity. Reproduced with permission.^[^ ^21^ ^]^ Copyright 2019, Wiley‐VCH.

PT‐OPDs with layered structure exhibit better photoresponsivity and detectivity because of efficient exciton generation and dissociation at the interfaces when applying illumination and bias electric field.^[^ ^23^, ^24^ ^]^ In 2018, Cheng et al. fabricated a voltage‐driven erasing nonvolatile memory based on PT‐OPDs, which utilized a novel aggregation enhanced emission‐active aromatic polyamide as a polymer electret layer. The fabricated devices showed high photoresponsivity and photosensitivity to UV irradiation, which was ascribed to the good light absorption of the pentacene channel.^[^ ^25^ ^]^ Stable erasing states driven by voltage was thus obtained. Similarly, Huang et al. introduced a highly fluorescent aggregation‐induced emission electret into PT‐OPDs to boost UV light response for the application of photomemory storage and photodetectors. As shown in **Figure** **2**a, the devices have a similar layered structure to that of Cheng's research.^[^ ^26^ ^]^ Under the UV light illumination, excitons can be generated in the pentacene layer. When a gate voltage was applied, excitons would be dissociated into electrons and holes. Then the charge separation could be realized while electrons were trapped into the electret layer, which could relatively increase the concentration of holes. The working mechanism could be seen clearly in Figure 2a. Based on the photogating effect, the best‐performing PT‐OPDs obtained an extraordinary photoresponsivity of 45 A W^−1^ and photosensitivity of 1.92 × 10^6^.

Qi et al. demonstrated high‐performance thermally stable PT‐OPDs using a donor material, PSeTPTI, which showed a wide light absorption spectrum from UV to visible region and could prolong the response time due to the existence of enormous electrons traps.^[^ ^27^ ^]^ By depositing PC_61_BM on the surface of donor film, as shown in Figure 2b, the photoresponsivity and response time could be largely improved because of the introduction of PSeTPTI/PC_61_BM heterojunction that facilitated photocurrent generation. The fabricated devices showed a significant photoresponsivity of 2 × 10^4^ A W^−1^ and detectivity of 3.1 × 10^16^ Jones (Figure 2b). Besides, hybrid organic‐inorganic perovskite materials were also explored to improve the property of PT‐OPDs. Luo et al. provided a new layered structure with the FAPbI_3_ perovskite and DNTT heterojunction to fabricate a hybrid phototransistor, which is shown in Figure 2c.^[^ ^21^ ^]^ Because of the small exciton binding energy, excitons could dissociate into holes and electrons more easily under light irradiation. While holes were diffusing to the DNTT layer, electrons would be trapped in the perovskite layer. The trapped electrons modulated the conductivity of the DNTT layer, which further accelerated the transportation and recirculation of holes. The devices showed a broadband photoresponse from the deep‐UV to the NIR region. Photoresponsivity and detectivity of 778 A W^−1^ and 1.04 × 10^13^ Jones were obtained, respectively (Figure 2c).

### 2.3 Blended Structure

**Figure 3:**
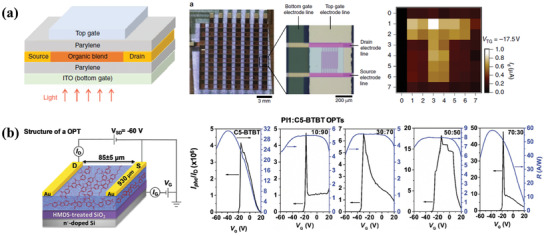
a) Schematic device structure of dual‐gate PT‐OPDs, an optical micrograph of the detector array, and the detected image. Reproduced under the terms of the CC‐BY 4.0 international license.^[^ ^32^ ^]^Copyright 2018, The Authors. Published by Springer Nature. b) Schematic diagram of the device structure, photosensitivity, and photoresponsivity of PI1:C5BTBT PT‐OPDs. Reproduced with permission.^[^ ^33^ ^]^ Copyright 2017, Wiley‐VCH.

Though great progress has been made in layered PT‐OPDs, some problems need to be tackled. A large number of excitons cannot dissociate efficiently and even recombine conversely due to the short exciton diffusion length, which is usually less than 10 nm for organic semiconductors. The performance improvement of PT‐OPDs is thus restricted due to insufficient charge collection. Blended donor and acceptor heterojunctions with the charge‐trapping effect are widely investigated because of the efficient exciton dissociation regardless of the limitation of exciton diffusion length. By using such structure, photocurrent generation is largely boosted and device performance is simultaneously increased. The combination of polymer donors with small‐molecule acceptor has been reported frequently.^[^ ^28^, ^29^, ^30^ ^]^ In 2014, Chen et al. proposed PT‐OPDs with a wide spectral response from UV to NIR light based on Cd_3_P_2_/PC_61_BM heterojunction. The enhanced device performance was ascribed to the formation of the junction interface and the favorable alignment of the conduction band that enhanced photoconductive gain and prolonged the carrier lifetime.^[^ ^31^ ^]^ Moreover, by introducing a dual‐gate field effect transistor based on an organic bulk‐heterojunction blend, PT‐OPDs with higher photoconductive gain and detectivity are demonstrated. Chow et al. reported a new device concept of dual‐gate phototransistor with the bulk heterojunction of MDMO‐PPV and PCBM blend, as shown in **Figure** **3**a.^[^ ^32^ ^]^ With this new device concept, both gates could accumulate and deplete both types of charge carriers to form conductive n‐ and p‐type channels at the dielectric‐semiconductor interface of gate. This could create an electric field equivalent to the built‐in field in diodes which helps to separate electrons and holes. Therefore, high gain and linear photoresponse could thus be achieved simultaneously without any external circuitry. On the other hand, a dual‐gate operation could also reduce the electrical noise, which led to three orders of magnitude improvement in detectivity. At last, they fabricated a 2D 8 × 8 arrays of dual‐gate PT‐OPDs for imaging, which provided a promising and practical application for highly sensitive photodetectors with tunable dynamic range.

Besides, Ljubic et al. fabricated a high‐performance, stable, and hysteresis‐free organic phototransistor, which consisted of polyimide (PI) and 2,7‐dipentyl‐benzothieno3,2‐b[1]benzothiophene (C5‐BTBT) as shown in Figure 3b. When the blend ratio was tuned to 70:30, the fabricated PT‐OPDs showed good Ohmic contact compared to other counterparts. Due to the strong electron‐withdrawing of PI, devices possessed an improvement of drain current in the dark and achieved a high photoresponsivity of 429 A W^−1^ and photosensitivity of 10^6^ under UV illumination. This work identified a facile and promising method for high‐performance OPDs.^[^ ^33^ ^]^

### 2.4 Composite Structure

**Figure 4:**
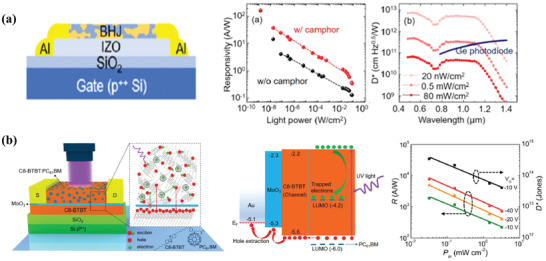
a) Phototransistor structure with indium zinc oxide (IZO) as an electron transporting layer. Photoresponsivity and detectivity as a function of incident light power. Reproduced with permission.^[^ ^34^ ^]^ Copyright 2019, American Chemical Society. b) Schematic device structure, energy level diagram, and photoresponsivity as a function of power intensity for hybrid‐layered PT‐OPDs. Reproduced with permission.^[^ ^35^ ^]^ Copyright 2019, Wiley‐VCH.

Though the advantages of blended PT‐OPDs, there are still some limitations to be further improved. For example, the existence of the donor/acceptor interface has a bad impact on trapped charges, which will increase the channel resistance and lower the carrier lifetime. The relatively high dark current impedes the photosensitivity and detectivity of PT‐OPDs. Furthermore, both layered structure and blended structure have a trade‐off between charge separation, charge transportation, and photon absorption. To tackle the problem, a novel PT‐OPD with the composite structure is proposed, which combines the advantages of layered PT‐OPDs and blended PT‐OPDs and achieves the improved carrier collection. In 2019, Kim et al. reported high‐performance PT‐OPDs based on a bilayer transistor channel with a structure of Si/SiO_2_/indium zinc oxide (IZO)/infrared‐sensitive polymer: PC_71_BM/Al (**Figure** **4**a).^[^ ^34^ ^]^ By putting the high‐permittivity molecule camphor into the bulk heterojunction (BHJ), the excitons binding energy of the organic BHJ layer was significantly changed. Photogenerated charges were increased with the prolonged carrier transporting length. Under the illumination of visible and NIR light with the wavelength from 500 to 1400 nm, the excitons would be generated and separated in the BHJ layer. Moreover, holes were trapped and electrons were circulated in the transport layer consisting of IZO, which had good electron mobility. Then the photoconductive gain was obtained. In this respect, the photogeneration and charges transport could be proceeded in different channels, which allowed independent optimization of each layer and resulted in a high detectivity of 5 × 10^12^ Jones and a dynamic range of 127 dB (Figure 4a). Similarly, Han et al. proposed an ultrahigh photosensitive PT‐OPDs with a broad spectral response (from 410 to 740 nm) and parallel structure of Si/SiO_2_/Octadecyltrichlorosilane (OTS)/FBT‐Th4(1,4):PC_61_BM/Au electrodes.^[^ ^20^ ^]^ With the trapping of electrons, the enhanced photocurrent and photoconductive gain were obtained, yielding an outstanding performance of a photoresponsivity of 1.2 × 10^5^ A W^−1^ and spectral detectivity of 3.18 × 10^16^ Jones at zero bias with 0.55 µW cm^−2^ at 700 nm.

Recently, Gao et al. reported a novel composite PT‐OPDs by taking advantage of both the charge‐trapping effect and efficient carrier transport with the structure of Si/SiO_2_/C8‐BTBT/MoO_3_/C8‐BTBT: PC_61_BM/Au, which can be seen in Figure 4b.^[^ ^35^ ^]^ Similar to the mechanism mentioned above, excitons could be generated and separated in the BHJ film under UV illumination, meanwhile, electrons could be trapped in the acceptors. C8‐BTBT was selected as the channel material due to its advantages of high mobility, excellent stability, and high solubility. The utilization of C8‐BTBT boosted the holes injection and increased photocurrent. Especially, an ultrathin MoO_3_ layer inserted into metal (or BHJ)/ C8‐BTBT interface showed great significance in the enhancement of device performance. It was summarized in these aspects: 1) to facilitate the holes injection and prevent electrons to recombine with holes after photogeneration and separation; 2) to decrease the contact resistance of metal/organic interfaces; 3) to impede the degradation of the channel layer. In this way, a great improvement in photodetection performance with a photoconductive gain was realized. Outstanding photosensitivity of 2.9 × 10^6^, a photoresponsivity of 8.6 × 10^3^ A W^−1^, and a detectivity of 3.4 × 10^14^ Jones were obtained. This strategy demonstrated a new insight into the design and optimization of high‐performance PT‐OPDs with a broad spectrum.

## 3 PC‐OPDs

### 3.1 Operation Mechanism

Unlike PT‐OPDs, PC‐OPDs consist of an organic semiconductor layer simply, which has only two electrodes attached to the opposite ends with the active layer by Ohmic contact. PC‐OPDs are designed to trap the minority charge carriers so that the majority charge carriers can recirculate through the device many times before recombination. That is to say, with the absorption of a photon, the generated photocarrier can be collected multiple times, in which case photoconductive gain and a high EQE exceeding 100% can be realized. Thus, PC‐OPDs can exhibit excellent performance through the PM effect.^[^ ^36^, ^37^, ^38^ ^]^

Theoretically, the PM effect can be excited by the holes/electrons tunneling injection at the interfaces.^[^ ^39^ ^]^ Corresponding devices need sufficient charge injection from the external circuit and enough charge transport under illumination while trapping the opposite charges in the active layer.^[^ ^40^ ^]^ As is known to all, the photosensitivity is a vitally important parameter to evaluate the performance of photoconductors, as well as gain. And photosensitivity is the ratio of the photocurrent generated to the dark current at fixed incident optical power and bias voltage conditions. Gain can be defined as a ratio of the output current of a photodetector to the current directly produced by the photons incident on the detectors. A high gain is required to amplify the signal far above the noise baseline in order to achieve high photosensitivity. Generally, higher EQE and lower dark current density will benefit the improvement of photosensitivity.^[^ ^41^, ^42^, ^43^ ^]^ PC‐OPDs are featured by high photosensitivity in consequence. According to the operation mechanism, there are also two modes in PM effect, which are hole trap‐assisted electron tunneling injection and electrons trap‐assisted holes tunneling injection.

### 3.2 Electron Tunneling Injection

In 1994, the PM effect was first proposed by Hiramoto et al. through an organic n‐type perylene pigment (Me‐PTC) with the structure of glass/Au/Me‐PTC/Au.^[^ ^44^ ^]^ Under an applied electric field, the PM effect could be realized in the interface between organic film and Au electrode. With electron tunneling injection assisted by trapped holes, 10 000 times improvement in photocurrent multiplication was obtained. Later, the PM effect was studied deeply with the effort of lots of researchers. In 2006, Howard and coworkers fabricated an infrared photoconductor by spin‐coating a prefabricated planar electrode array with an unpatterned layer of PbS colloidal quantum dot nanocrystals.^[^ ^36^ ^]^ Under the effect of surface trap states, the photoconductive gain was achieved, which showed a photoresponsivity of higher than 10^3^ A W^−1^ and a normalized detectivity of 1.8 × 10^13^ Jones at room temperature.

**Figure 5:**
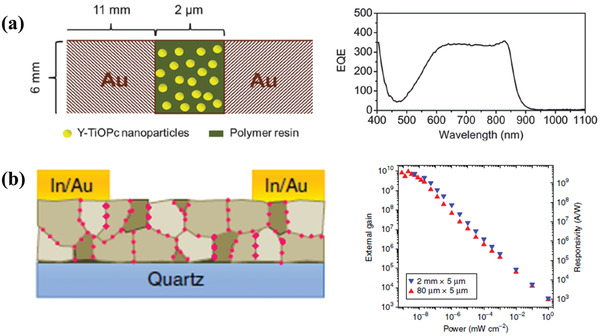
a) Schematic device structure and EQE spectrum of a NIR photoconductor. Reproduced with permission.^[^ ^39^ ^]^ Copyright 2015, The Royal Society of Chemistry. b) Schematic device structure, external photoconductive gain, and photoresponsivity of the doped photoconductor. Reproduced under the terms of the CC‐BY 4.0 international license.^[^ ^19^ ^]^ Copyright 2019, The Authors. Published by Springer Nature.

In 2015, Peng et al. exhibited a highly sensitive NIR photoconductor with the structure of Au/Y‐form oxotitanium phthalocyanine nanoparticles: polycarbonate resin/Au, which showed a broad spectral response range (**Figure** **5**a).^[^ ^39^ ^]^ Owing to the holes trapping effect in the interfaces under illumination, efficient electrons tunneling occured with the realization of photoconductive gain, which resulted in a brilliant EQE value of 35 600% under the illumination of 830 nm light. In the meanwhile, Zhang et al. provided a new photoactive material to reduce the recombination of electrons and holes, which was fabricated by sintering chloride‐capped CdTe nanocrystals into polycrystalline films, where Cl selectively segregated into grain boundaries acting as n‐type dopants (Figure 5b).^[^ ^19^ ^]^ Photogenerated electrons concentrated in and percolated along the grain boundaries, while holes were confined in the grain interiors. Therefore, the photoconductor could exhibit a high gain of 1 × 10^10^ and an incomparable photoresponsivity of 4 × 10^9^ A W^−1^ at the wavelength of 500 nm.

### 3.3 Hole Tunneling Injection

Holes tunneling injection assisted by trapped electrons has also made great progress under the effort of researchers. Zhang and coworkers demonstrated OPDs with an extraordinary performance through materials synthesis,^[^ ^45^, ^46^ ^]^ and structure design of active layers,^[^ ^17^, ^47^, ^48^ ^]^ which have EQE value higher than 100% at different wavelength due to the PM effect.

To increase the performance of PC‐OPDs, such as photocurrent, photoresponsivity, and detectivity, many researches have been carried out. Li et al. successfully improved the photocurrent of the P3HT:PCBM PC‐OPD using a substrate with wavelength‐matched patterned photonic crystals, which could be put down to the efficient reflection of particular light and the defects from the rough structure of photonic crystals.^[^ ^49^ ^]^ Later, Jin et al. fabricated a PIN architecture‐based PC‐OPD with the device structure of Al/P3HT/P3HT:PCBM/PCBM/Al.^[^ ^50^ ^]^ Benefitting from the proposed PIN architecture, increased hole mobility was obtained, which further resulted in a high responsivity of 96.5 A W^−1^.

**Figure 6:**
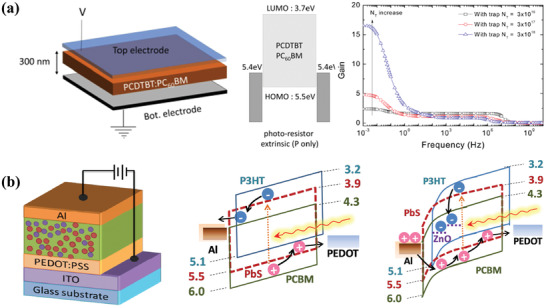
a) Schematic device structure, energy diagram, and gain dependency versus frequency with different trap concentrations of PC‐OPDs.Reproduced with permission.^[^ ^52^ ^]^ Copyright 2020, AIP Publishing. b) Schematic device structure and work mechanism of hybrid PD‐OPDs. Reproduced with permission.^[^ ^54^ ^]^ Copyright 2014, Wiley‐VCH.

Furthermore, Chaudhary et al. investigated the barrier potential reduction and photoresponse improvement based on different ratios of P3HT:DH6T blends in PC‐OPDs.^[^ ^51^ ^]^ After optimization, an improvement of the morphology near to metal‐polymer interface and a reduction in hole injection barrier were obtained, which led to a higher photocurrent in PC‐OPD. Additionally, Hirsch and coworkers analyzed the mechanism of the high‐level gain and EQE of vertical PC‐OPD (**Figure** **6**a). Based on P only photoconductors, photogenerated holes could cause additional carrier injection from the contacts when a significant modification of electric field was induced, which was called “gain by injection enhancement.”^[^ ^52^ ^]^

To the best of our knowledge, PC‐OPDs can achieve a high gain by the Ohmic contact with one electrode, however, they also show a large dark current due to the large charge injection and the relatively smaller shunt resistance. To solve the issue, a combination of PC‐OPD with high gain and PD‐OPD with low noise is put forward. In 2012, Guo et al. fabricated a highly sensitive UV photodetector with a vertical structure, which possessed an active layer of polymer (PVK for UV detection or P3HT for UV‐Vis detection): ZnO nanocomposite and combined the photoconductive gain of PC‐OPDs and the low noise of photodiodes.^[^ ^53^ ^]^ In the dark, electrons were trapped in the nanoparticles due to the strong electron trapping effect, which showed a low dark current for the very large charge injection barrier. Under illumination, the trapped electrons could quickly transport and align the Fermi energy of the nanocomposite with that of the cathode, which made the hole‐injection barrier on the cathode so thin that the holes could easily tunnel through it with small reverse bias. Based on this, a photoconductive gain was thus obtained with a high EQE of 340 600% and a great photoresponsivity of 1001 A W^−1^. Meanwhile, the low dark current was also achieved. Later, Dong et al. provided a dual‐quantum dots hybrid photodetector, which was acting as a photodiode in the dark with Schottky contact and a photoconductor under illumination with Ohmic contact (Figure 6b).^[^ ^54^ ^]^ When putting lead sulfide and ZnO quantum dots into P3HT:PCBM blends, there would be a photoconductive gain under reverse bias and illumination, which was the same as the mechanism mentioned above. In this respect, the photodetector demonstrated a broad spectral response and a good property.

## 4 PD‐OPDs

### 4.1 Operation Mechanism

Organic photodiodes are the diodes with a light‐sensitive depletion region in which incident photons generate electron‐hole pairs through the photoelectric effect. Benefiting by the effect of the electrical polarization, electrons diffuse toward the cathode and holes diffuse to the anode, which creates a reverse photocurrent within the photodiodes (from the cathode to the anode) that is linearly proportionate to the flux of incident light. The photodiodes can operate in two detection modes: photovoltaic (PV) mode and photoconductive (PC) mode, which is determined by the organic photodiodes under reverse biased or zero biased. When the photodiodes are reverse biased, the electrical polarization of the depletion region becomes broader and more sensitive to photocurrent generation, meanwhile, the generated reverse photocurrent can be output from the photodiodes, which is called PC mode. In contrast, when the photodiodes are zero biased, the electrical polarization is not strong enough that the flow of photocurrent will be suppressed. At this time, the internal reverse photocurrent makes electrons and holes accumulate at the cathode and anode, respectively, which forms a photovoltage. Thus, organic photodiodes are operated in PV mode while the photodiodes are not reverse biased.^[^ ^11^, ^55^ ^]^ Due to the inevitable charge recombination, the EQE of PD‐OPDs is lower than that in phototransistors and photoconductors. For example, Gasparini et al. fabricated a highly efficient NIR organic photodiodes with a record responsibility of 0.42 A W^−1^ and EQE of 69% at 755 nm. Moreover, though organic photodiodes show a lower EQE of lower than 100%, they generally have low dark current, fast response, and wide linear dynamic range.^[^ ^16^, ^56^, ^57^, ^58^, ^59^, ^60^, ^61^, ^62^ ^]^

**Figure 7:**
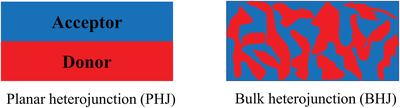
Schematic diagram of planar heterojunction and bulk heterojunction.

Since the first OPDs were fabricated with the organic small molecule dyes merocyanine and rhodamine B by Kudo and Moriizumi in 1981,^[^ ^63^ ^]^ which showed a spectral response in the visible spectrum range. Increasing research endeavors have been witnessed in extending spectral response and improving the sensitivity of OPDs. It is well known that the photocurrent is related to exciton dissociation, charge transportation, and charge collection. Hence, a general method to effectively separate electron‐hole pairs is put forward by forming a heterojunction between donor and acceptor materials. A driving force is provided for charge separation by introducing differing electron affinity and ionization potentials.^[^ ^64^ ^]^ According to the specific structure, the absorber can be divided into planar heterojunction (PHJ) and bulk heterojunction (BHJ), as is shown in **Figure** **7**. BHJ possesses an interpenetrating network of donor and acceptor materials with nanoscale phase segregation. In the PHJ‐based active layer, excitons can be separated quickly when reached the interface of donor and acceptor via diffusion. Nevertheless, the short exciton diffusion length that is regarded as 10 nm universally limits the increase in photocurrent.^[^ ^65^, ^66^ ^]^ BHJ structure is thus proposed to harvest sufficient excitons without sacrificing light absorption and shorten the average diffusing distance to junctions for excitons. A summary of recent progress in PD‐OPDs based on PHJ and BHJ structures is shown in **Table** **1**. To break the EQE limitation, PM effect has been introduced to PD‐OPDs by rational engineering of active layer. The operation mechanism of PM‐type PD‐OPDs is similar to PC‐OPDs, while their device structure and materials selection are different. PM‐type PD‐OPDs exhibit a structure of anode/hole transporting layer (HTL)/active layer/ electron transporting layer (ETL)/cathode in conventional type, or anode/ETL/active layer/HTL/cathode in inverted type, in which the work function of anode and cathode is different in order to facilitate the collection of photogeneration carriers. While PC‐OPDs show a structure of electrode/active layer/electrode, in which the two electrodes have similar work functions. However, it also has some negative influences on other device performance in virtue of unbalance charge collection.

**Table 1:** Some performance parameters of OPDs with different active layers

	Active layer	EQE [%]	R [A W^−1^]	D [Jones]	Ref
PHJ	TAPC:C_70_	56.0	0.144	2. 5 × 10^13^	^[^ ^67^ ^]^
PHJ	PbPc:C_70_	30.2	–	4.2 × 10^12^	^[^ ^68^ ^]^
PHJ	P3HT:PCBM	–	–	1.3 × 10^12^	^[^ ^69^ ^]^
PHJ	ZnO:PPDT2FBT	0	0.150	3.0 × 10^12^	^[^ ^70^ ^]^
PHJ	Poly‐C_60_:Cy7‐T	23.0	0.160	1.0 × 10^12^	^[^ ^71^ ^]^
BHJ	Co1‐4Cl:PTB7‐Th	68.0	0.500	1.0 × 10^12^	^[^ ^72^ ^]^
BHJ	PbPc:C_60_	31.1	–	9.0 × 10^12^	^[^ ^73^ ^]^
BHJ	PbS‐QD:P3HT:PCBM	51.0	0.500	2.3 × 10^9^	^[^ ^74^ ^]^
BHJ	NPB:OXD‐7	–	0.023	1.0 × 10^12^	^[^ ^61^ ^]^
BHJ	EHTPPD‐MT:PC_61_BM	20.0	0.175	–	^[^ ^75^ ^]^
BHJ	PC_71_BM:CPDT‐*alt*‐BSe	–	–	1.0 × 10^12^	^[^ ^76^ ^]^

### 4.2 Planar Heterojunction Structure

**Figure 8:**
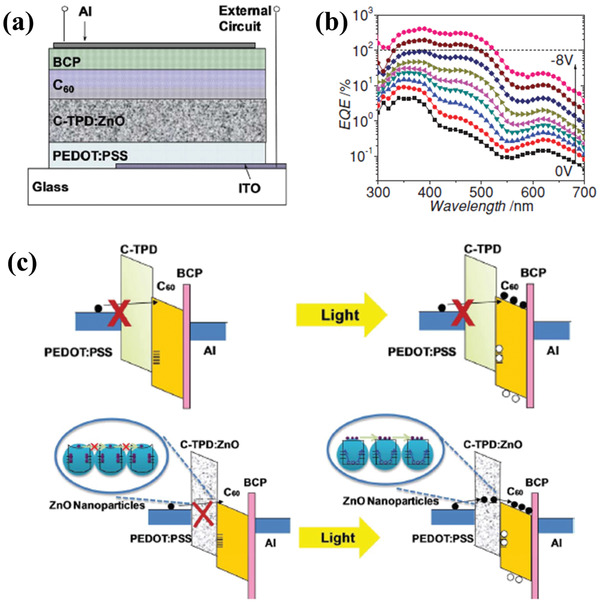
a) Schematic device structure of the photodetector with ZnO nanoparticles mixed in the C‐TPD buffer layer. b) EQE spectra of the photodetector under reverse bias from 0 to −8 V with a voltage step of 1 V. c) Energy level diagram of the reverse‐biased photodetector in dark and under illumination. Reproduced with permission.^[^ ^79^ ^]^ Copyright 2014, Wiley‐VCH.

Planar heterojunction structure has been developed for many years, which shows a similar operation mechanism to layered PT‐OPDs in terms of photoactive layers. Recently, Zhu et al. built a self‐powered UV photodetector combining a mesoporous TiO_2_ bilayer and a Spiro‐OMeTAD layer. The nanostructured heterointerface was engineered by organic modifiers.^[^ ^77^ ^]^ By using such a method, the photoresponsivity was boosted by 70% with a photoresponsivity of 64 mA W^−1^ at 380 nm and a very high sensitivity of exceeding 10^4^. C_60_ was used because of its excellent optoelectronic property with high electron mobility. Huang and coworkers used C_60_ to trap holes and reduce the electron injection barrier of photodetectors. The device performance was largely optimized with a detectivity of 3.6 × 10^11^ Jones at 370 nm.^[^ ^78^ ^]^ To continuously promote the properties of OPDs, ZnO nanoparticles were inserted into C‐TPD as a buffer layer to fabricate ultraviolet photodetectors, which used the structure of ITO/PEDOT:PSS/C‐TPD:ZnO/C_60_/BCP/Al,^[^ ^79^ ^]^ as shown in **Figure** **8**a. In Figure 8b, it was found that the peak EQE values were higher than 100% at the reverse bias of −6 V at 390 nm and further increased to 408% when the reverse bias was −8 V. According to the relatives between responsivity and EQE, the corresponding responsivity was calculated to be 1.28 A W^−1^ at 390 nm, which was five times large than those in commercial SiC and GaN UV photodetectors. The energy band diagrams were given to understand the underlying mechanism of the enhancement of device performance (Figure 8c). The C‐TPD layer would prevent electrons injecting from PEDOT:PSS to C_60_ layer due to the low electron mobility and the large electron injection barrier. For devices with ZnO nanoparticles, electrons could transport through ZnO nanoparticles and then be injected into the C_60_ layer without high resistance. Moreover, ZnO nanoparticles could help to trap photon‐generated holes, which could cause a high gain and photocurrent.

### 4.3 Bulk Heterojunction Structure

Unlike PHJ structure, BHJ structure features efficient exciton dissociation and extraction, which breaks the limitation of the exciton diffusion length. Based on BHJ structure, many advances have been achieved in OPDs with a detecting wavelength from UV to NIR. In 2015, Lee et al. fabricated PD‐OPDs with all‐small molecular BHJ active layers, in which EHTPPD‐MT and PC_61_BM were used as electron donor and acceptor, respectively. The PD‐OPDs exhibited broad optical absorption range from 300 to 800 nm and stable responses under modulation of repeated incident light with 75–180 mA W^−1^ at 532 nm and 80–170 mA W^−1^ at 650 nm from 0 to −1 V, respectively.^[^ ^75^ ^]^ Later, Lee et al. synthesized two narrow bandgap electron acceptors, CTIC‐4F and CO1‐4F, which had good absorption in the 700–1100 nm range. When blending with PTB7‐Th, efficient OPDs were obtained with responsivities of 0.51 A W^−1^ at 830 nm and 0.52 A W^−1^ at 920 nm for CTIC‐4F and CO1‐4F, respectively.^[^ ^80^ ^]^

**Figure 9:**
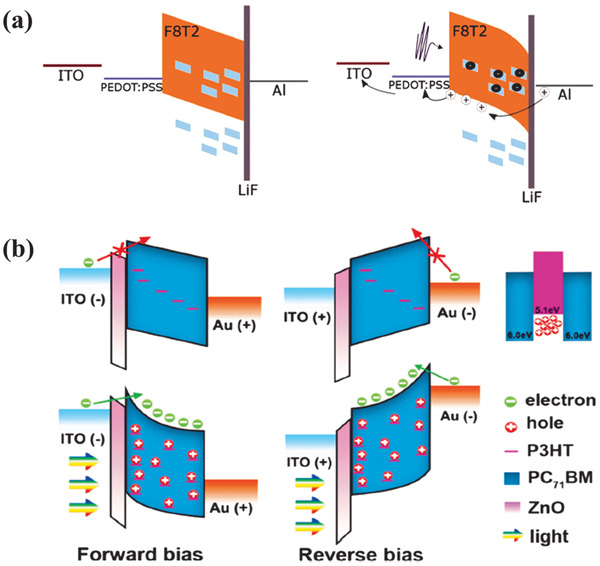
a) The relative band energies of the device with and without illumination. Reproduced with permission.^[^ ^82^ ^]^ Copyright 2017, Wiley‐VCH. b) Schematic band structure of the interfacial band bending in PM type OPDs under the different situations of illumination and bias. Reproduced with permission.^[^ ^85^ ^]^ Copyright 2020, The Royal Society of Chemistry.

Meanwhile, the ratio of donor to the acceptor in the active layer has been adjusted to an extraordinary value to trap minority charges and generate the PM effect of the majority charges. The significant breakthrough in EQE of PD‐OPDs was thus realized. Ryu and coworkers fabricated all‐organic UV photodetectors with a high gain and an EQE of 25 300% on account of the introduced electrons tunneling injection by trapped holes. The device showed a high EQE and detectivity even after the exposure for 50 days.^[^ ^81^ ^]^ In 2017, Calcagno and coworkers fabricated ultraviolet OPDs with the structure of ITO/PEDOT:PSS/F8T2:PC_71_BM/LiF/Al, which the weight ratio of the active layer was 100:4. The devices exhibited low dark current density and high sensitivity.^[^ ^82^ ^]^ They also found that EQE was increased gradually with the increase of reverse bias, showing a maximum value of 5600% at 360 nm. To clearly understand the enhancement mechanism, the energy level diagrams were given in **Figure** **9**a. Regardless of the reverse bias applied in the dark, no or fewer charges were transporting between layers because of the high energy barrier, which guaranteed the relatively low dark current density. Under light illumination and reverse bias, electrons were trapped in PC_71_BM because of the high barrier of 1.2 eV, while the holes could be injected into F8T2 easily due to the band bending. Photomultiplication was obtained when majority carriers were collected, resulting in a high EQE. A similar effect could be seen in Miao's and Wang's works, which achieved a maximum of 10 600% and 3660% in EQE, respectively.^[^ ^83^, ^84^ ^]^

Recently, Zhang and coworkers successfully fabricated PM type organic photodetectors with the structure of ITO/ZnO/PC_71_BM:P3HT/Au, in which the ratio of the concentration of the active layer was 100:5 (Figure 9b).^[^ ^85^ ^]^ Under the different situations of illumination and bias, this PM type OPDs exhibited excellent performance with an EQE value of 3900% at 355 nm under 5 V bias and 4900% at 640 nm under −5 V bias. In the dark, electrons could not transport into the active layer due to the large energy barriers. While under illumination, holes were trapped in P3HT, and interfacial band bending was formed, which made electrons transport more easily from the external circuit.

### 4.4 Hybrid Structures

In addition to use organic materials as the active layer with PHJ or BHJ structures, photodiodes based on hybrid organic and inorganic nanocomposites also offer a high property in sensitivity and detectivity, such as hybrid inorganic nanoparticles or quantum dots. For example, in 2014, Dong et al. provided a quantum dots hybrid photodetector with EQE higher than 100%.^[^ ^54^ ^]^ Li et al. successfully fabricated NIR photodiodes by mixing a Y‐type titanylphthalocyanine nanoparticles (Y‐TiOPc NPs) and obtained the highest EQE value of 354 200% and excellent photosensitivity of 2227 A W^−1^.^[^ ^40^ ^]^

**Figure 10:**
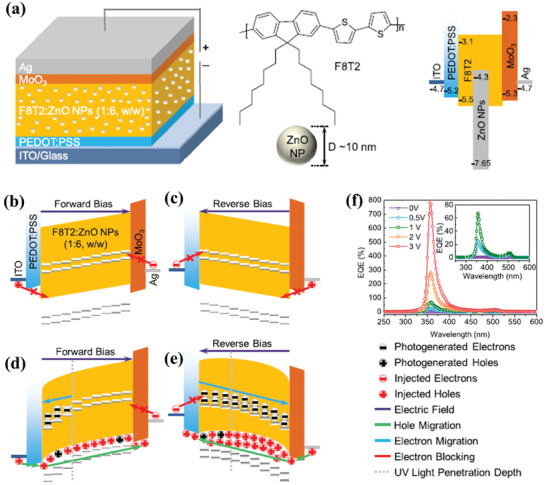
a) Device structure, active layer materials structure, and energy band diagram of UV photodetectors. Illustration of the device in dark under b) forward bias and c) reverse bias. Illustration of the device with illumination under d) forward bias and e) reverse bias. f) EQE spectra of the device under different forward bias. Reproduced with permission.^[^ ^86^ ^]^ Copyright 2018, American Chemical Society.

Recently, Zheng et al. employed nanocomposite thin films consisting of F8T2 and ZnO nanoparticles (NPs) as an active layer with a weight ratio of 1:6 to fabricate UV photodetectors.^[^ ^86^ ^]^ The main purpose of introducing ZnO NPs was to provide electron traps, which could cause energy band bending for holes injection and realize PM phenomenon. It was suggested that this structure favored hole transport than electron transport due to the same work function of two electrodes and the similar highest occupied molecular orbital between PEDOT:PSS and MoO_3_. The device structure of UV photodetectors and the energy band were shown in **Figure** **10**a, and the working mechanism under forward or reverse bias was illuminated in Figure 10b–e. The holes injection was blocked by the energy barriers between ITO and F8T2 under forward bias in the dark, meanwhile, electrons injection was also blocked by the MoO_3_ layer, which led to a low dark current density. Under a reverse bias in the dark, a lower dark current could be obtained by the same blocked charges because of the energy barriers. When applied light illumination and a forward bias, excitons could be generated and then separated between the interface of F8T2 and ZnO NPs, in which case holes could be transported into Ag electrode and electrons would be trapped in ZnO NPs due to the large energy barriers that made electrons transport pathway disrupted. With the accumulated number of electrons, the energy band closed to the PEDOT:PSS side would be bend, which facilitated holes injection and made photocurrent density 10^5^ orders of magnitude higher than the dark current density. As a result, a high EQE value of 782% was attained under a low forward bias of 3 V at 358 nm, and a correspondingly high detectivity was also obtained of 8.45 × 10^12^ Jones, as shown in Figure 10f.

## 5 Spectrally Selective Detection

As is well‐known, the light response of OPDs varies from the wavelength. The relationship between photosensitivity and wavelength is called the spectral response characteristic, which commonly has a spectral response range from 200 to 1100 nm. OPDs can realize different spectral responses through selecting the materials of active layers, which have diverse absorption spectra with the wavelength range from ultraviolet to near‐infrared. Based on this, photodetectors show a big difference in the detection range of single wavelength region or broad wavelength region from UV to NIR.^[^ ^31^ ^]^ To date, the progress is implemented in narrowband and broadband single‐mode OPDs through the design and selection of active layers, which also shows flexibility,^[^ ^87^ ^]^ high thermal stability,^[^ ^27^, ^75^ ^]^ fast response,^[^ ^88^ ^]^ high sensitivity, and so on.^[^ ^62^, ^89^ ^]^

### 5.1 Narrow‐Band Detection

Organic semiconductors exhibit the ability to tailor the spectral response range by modifying its chemical structure. They are usually used as active layers in solar cells because of their broadband absorption range that enables the maximal sunlight harvesting. As for OPDs, the broadband response can be easily achieved based on active layers materials with broad absorption range from UV to NIR. In contrast, it is challenging to realize narrowband OPDs. Many efforts have been made in accomplishing narrowband detection range in OPDs in recent years. The most traditional method to obtain narrowband photodetectors is combining bandpass filters with broadband photodetectors. Through this strategy, the color selectivity in CMOS image sensors can be realized, but the use of color filters will make the fabrication more complicated and also reduce the detectivity due to the attenuating of the incident light.^[^ ^16^ ^]^ Therefore, other strategies should be put forward to realize the narrowband detection. First, making full of narrowband absorbers by suitable design of organic materials is an efficient way to achieve this goal. OPDs with a narrowband detection between UV and NIR are widely reported, which also can exhibit high performance in photosensitivity and detectivity. In 2017, Li et al. provided a narrowband green‐light organic photodiode employing squarylium material with the structure of donor–acceptor–donor (D–A–D), which showed a narrow response spectrum with a full‐width at half‐maximum (FWHM) of 110 nm and high EQE of 60% in green region at −2.5 V. Meanwhile, the device exhibited a very low dark current of 5.4 nA cm^−1^ and high specific detectivity of 7.7 × 10^12^ Jones.^[^ ^90^ ^]^ In 2019, Xia et al. employed the electron acceptor, SBDTIC, to bulk‐heterojunction narrowband detection OPDs, which showed a spectrally suitable absorption peak at 730 nm for far‐red photodetection. When combined with the transparent donor (PolyTPD) the fabricated devices exhibited an excellent narrowband functionality with a peak specific detectivity of 1.42 × 10^13^ Jones and a spectral width of 141 nm in self‐powered mode, in which such OPDs also showed a response with the microsecond range and a quasilinear response over a photocurrent range of least four orders of magnitude.^[^ ^91^ ^]^

Besides, a more promising strategy for OPDs with narrowband detection is to manipulate internal quantum efficiency via charge collection narrowing (CCN).^[^ ^92^, ^93^ ^]^ The working principle of CCN connects with the wavelength‐dependent absorption coefficients of incident photons within the photoactive layer. According to Beer–Lambert law, photons with an energy matching the absorption peak of materials will be absorbed in the surface of the active layer. Due to the low charge mobility and high thickness of active layer, the generated charges tended to recombine before reaching the back electrodes.^[^ ^1^ ^]^ Through the EQE spectra at −0.5 V of optimized red, green, and blue narrowband photodiodes and related junction absorption coefficients, the desired photoresponse and the blindness outside their designed spectral window were achieved, which clearly showed the realization of the CNN concept across the visible spectrum.

In contrast, photons with energy near the absorption tail can permeate further into active layer and charges can be generated uniformly across the thick film, which leads to a more balanced charge collection at the front and back electrodes. Thus, a larger photocurrent and EQE are obtained at the wavelength near the absorption tail. In 2015, Armin et al. first developed high‐performance CCN OPDs by using a BHJ active layer with a thickness of 2 µm. The fabricated red‐selective and NIR‐selective devices achieved a narrow full‐width at half‐maximum (FWHM) of about 90 nm. Due to the reduced defect density of the thick BHJ film, the optimized narrowband OPDs demonstrated a maximum EQE of 30% and a high detectivity exceeding 10^12^ Jones.^[^ ^94^ ^]^ By tuning the spectral response, in the meanwhile, they also reported the narrowband red, green, and blue photodiodes without the use of color filters.^[^ ^95^, ^96^, ^97^ ^]^ Using the CCN concept in organohalide perovskites or mixed lead halides as solution‐processable semiconductors, the device exhibited truly filterless narrowband detection with FWHM of less than 100 nm.

**Figure 11:**
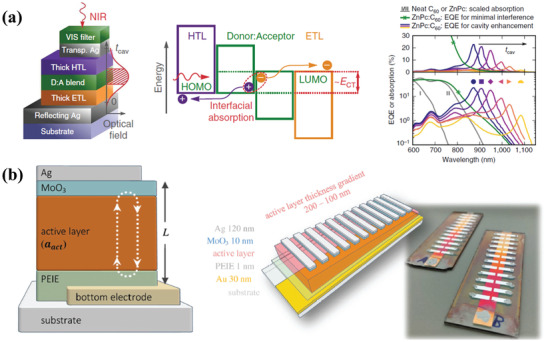
a) Schematic device structure, energy level diagram, and spectrally resolved EQE with the absorption of NIR OPDs. Reproduced under the terms of the CC‐BY 4.0 international license.^[^ ^99^ ^]^Copyright 2017, The Authors. Published by Springer Nature. b) The schematic device structure of resonant‐cavity‐enhanced OPDs and a photograph of a proof‐of‐concept miniature spectrometer. Reproduced with permission.^[^ ^100^ ^]^ Copyright 2017, Wiley‐VCH.

Also, there is another way to obtain a narrowband detection, which is to incorporate a resonant optical microcavity structure.^[^ ^98^ ^]^ In 2017, Leo's group successfully proposed a narrow‐band NIR OPDs with the active layer of ZnPc:C_60_ blends by using a resonant optical microcavity device.^[^ ^99^ ^]^ The schematic device structure and the energy levels diagram were exhibited in **Figure** **11**a. By introducing the optical microcavity architecture, the photocurrent and optical field could be enhanced within the intermolecular charge‐transfer absorption band, which increased the EQE slightly with the value of 20%. Moreover, the NIR OPDs also showed a broad spectral tunability from 810 to 1550 nm by changing the resonant wavelength. At the same time, they also fabricated wavelength‐tunable photodetectors with solution‐processed organic PBTTT:PCBM blends with similar microcavity structures (Figure 11b).^[^ ^100^ ^]^ After optimization, the charge‐transfer absorption could be enhanced, which enabled the EQE values higher than 20%. What is more, the miniaturized spectrometers were made by employing an active layer with gradient thickness, which demonstrated a new innovative area of photodetectors with the advantages of organic materials.

### 5.2 Broad‐Band Detection

**Figure 12:**
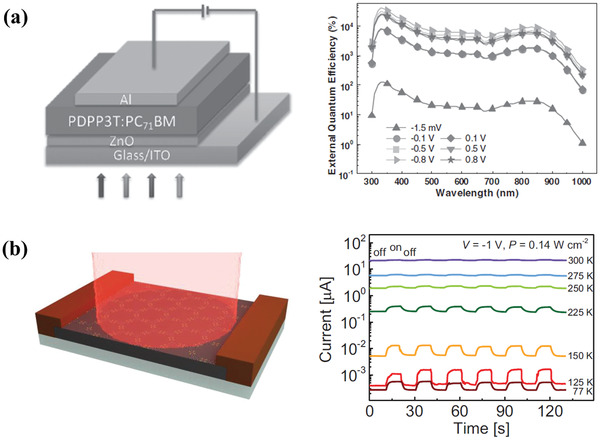
a) Device structure of the broadband photodiode and its EQE spectra measured under different bias voltages after UV light treatment. Reproduced with permission.^[^ ^56^ ^]^ Copyright 2016, Wiley‐VCH. b) Schematics of a two‐terminal photodetector device based on 1.7 µm thick MOF layer and temperature‐dependent photoresponse as a function of time at *P* = 0.14 W cm^−2^ and *V* = −1 V. Reproduced with permission.^[^ ^104^ ^]^ Copyright 2020, Wiley‐VCH.

Compared with narrowband detection in OPDs, it is much easier to realize broadband detection by using the active layer materials with a broad response range. Based on this, there are many applications in imaging sensors, optical communication, and so on. Many achievements have been reported in broadband detection, which utilizes the broad‐response organic material,^[^ ^54^, ^64^ ^]^ microwires or nanowires,^[^ ^31^, ^88^ ^]^ special nanostructures,^[^ ^21^, ^101^ ^]^ as well as hybrid organic and inorganic thin films.^[^ ^87^ ^]^ In 2016, Zhou et al. fabricated an ultrahigh gain polymer photodiode with a broad response from UV to NIR wavelength, which had a structure of ITO/ZnO/PDPP3T:PC_71_BM/Al (**Figure** **12**a).^[^ ^56^ ^]^ The device showed a large EQE of 140 000% and a broad response range from 300 to 1000 nm on account of the UV light introduced oxygen desorption and the increased current injection.

Besides, Chen et al. reported a hybrid organic‐inorganic heterojunction with PCBM and Cd_3_P_2_ nanowires. They first fabricated the phototransistors with single‐crystalline Cd_3_P_2_ nanowires by chemical vapor deposition methods, in which a largely spectral photoresponse was obtained with a broad wavelength from 300 to 1300 nm.^[^ ^31^ ^]^ Based on this, they demonstrated a hybrid phototransistor using a rigid and flexible substrate, which showed a better photocurrent and an improved photoconductive gain with a broad spectral range. Besides, carbon nanotubes (CNTs) can also be used in phototransistors with broad‐band detection due to their great potential in photonic and electronic applications, such as high tensile strength and resiliency, tunable spectral response, etc. Arnold et al. fabricated a broad‐band photodetector with a planar heterojunction of semiconducting polymers and electron acceptor, C_60_, in which the photogenerated excitons could be separated efficiently.^[^ ^64^ ^]^ When CNTs were wrapped in polymer, the relatively small offset of 0.2 eV between the interfaces of polymer wrapped by CNTs and C_60_ made it easier in the dissociation of generated excitons, which resulted in a better performance with detectivity of 10^10^ Jones from 400 to 1450 nm and a response time of 7.2 ± 0.2 ns. The combination of CNTs, polymer donors, and organic small molecules provided a facile way to realize the broader spectral responsivity.

Also, metal‐organic frameworks (MOFs) are a new class of porous and highly crystalline hybrid materials, which show a great attraction in optoelectronic applications due to their high tunability in physicochemical properties and fascinating architectures. However, there is still a challenge in optoelectronics with their poor electrical and high porosity, which can be improved by the suitable design of the constituents.^[^ ^102^ ^]^ Chen and coworkers first applied a crystalline MOF in graphene‐based OPDs and obtained ultrahigh photosensitivity and EQE on account of the outstanding mobility of graphene and the efficient photon trapping of porous MOF layer. Due to the inherent tunability and the broad spectral absorption of the MOF, OPDs could achieve a broadband detection from 325 to 700 nm.^[^ ^103^ ^]^ In 2020, Arora et al. demonstrated an optimized two‐terminal photodetector device with the active element of 2D MOF films of Fe_3_(THT)_2_(NH_4_)_3_, which operated a broad spectral range of 400–1575 nm (Figure 12b).^[^ ^104^ ^]^ It was found that photoresponse depended on the temperature, incident laser power, and wavelength. Due to the suppression of thermally activated charge carriers at liquid nitrogen temperatures (77 K), there was a dramatic improvement in responsivity and detectivity of 7 × 10^8^ Jones, which showed a promising future in the optimization of device structure and performance for photodetectors based on MOF films.

### 5.3 Dual‐Mode Detection

In addition to the single‐mode detection with narrowband or broadband detection, dual‐mode with multiple detections OPDs are also widely investigated. The spectral tunable OPDs possess practical interests without flipping the detectors, which broaden their application in wellness, security monitoring, and so on. In 2018, Zhang's group reported an organic photodetector with the structure of ITO/PEDOT:PSS/P3HT:PC_71_BM/Al.^[^ ^47^ ^]^ For binary OPDs, the OPDs exhibited narrowband response from 620 to 700 nm under the bottom illumination condition and broadband response from 300 to 700 nm under top illumination condition, respectively, which showed great potential in integrated optoelectronics products. In the meanwhile, they also provided photomultiplication type OPDs with the configuration of ITO/PFN‐OX/P3HT:PTB7‐Th:PC_61_BM/Al. With the help of the active layer, these OPDs could exhibit a tunable spectral response with broadband spectral response from 350 to 800 nm under forward bias and narrowband spectral response from 750 to 850 nm under reverse bias, which broadened the prospective application of OPDs.^[^ ^17^ ^]^

**Figure 13:**
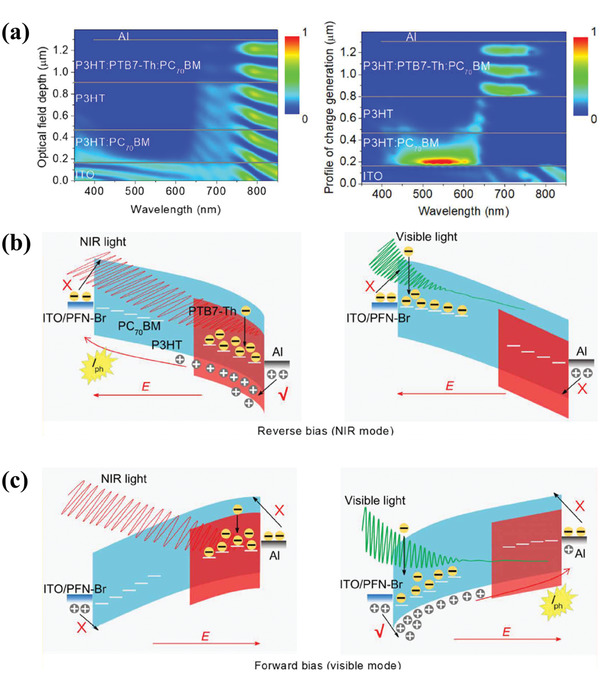
a) Simulated optical field distribution and photogenerated electron distribution in the device. The working principle of dual‐mode OPDs b) at a forward bias in the visible detection mode and c) at a reverse bias in the NIR detection mode. Reproduced with permission.^[^ ^105^ ^]^ Copyright 2020, American Association for the Advancement of Science.

Recently, Zhu's group reported a dual‐mode OPDs in NIR and visible light based on a bias‐switchable spectral response.^[^ ^105^ ^]^ By using a trilayer configuration of ITO/PFN‐Br/P3HT:PC_70_BM (100:1)/P3HT/ P3HT:PTB7‐Th/PC_70_BM (70:30:1)/Al, the device had a different light absorption in the two active layers, which corresponded to the simulated optical field distribution and photogenerated electron distribution at the wavelength range of NIR and visible light (**Figure** **13**a–c). Meanwhile, the P3HT layer was acting as an optical spacer to exhaust visible light and guarantee long‐wavelength light to reach the rear active layer. Based on these, the dual‐mode OPDs could be accomplished under forward and reverse bias with a brilliant property, which benefited from the PM effect above. This bias‐switchable OPDs under dual‐mode guided an attractive position in realizing the more unique and practical applications.

## 6 Flexible and Wearable OPDs

**Figure 14:**
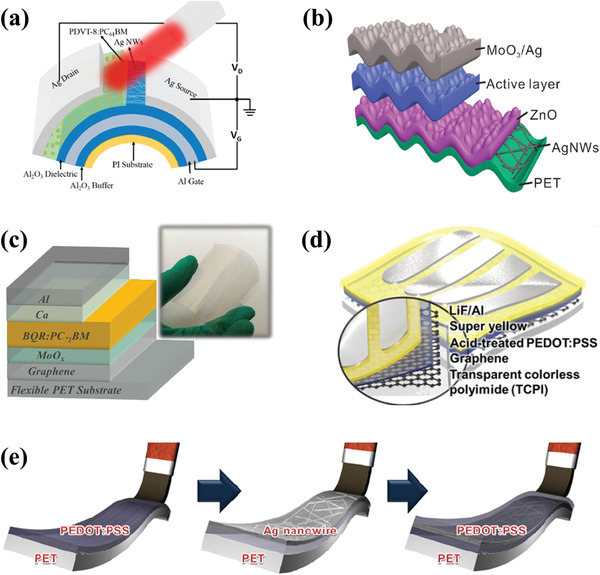
a) Device structure of flexible PT‐OPDs with ultrashort channel length. Reproduced with permission.^[^ ^110^ ^]^ Copyright 2018, American Chemical Society. b) Schematic of a flexible OSC with moth‐eye patterned AgNWs/ZnO flexible TCE. Reproduced with permission.^[^ ^115^ ^]^ Copyright 2019, American Chemical Society. c) Schematic and photograph of OSC with graphene electrode. Reproduced with permission.^[^ ^117^ ^]^ Copyright 2019, Wiley‐VCH. d) The schematic device structure of OSC with composite flexible TCE. Reproduced with permission.^[^ ^118^ ^]^ Copyright 2019, American Chemical Society. e) Fabrication progress of PEDOT:PSS/AgNWs/PEDOT:PSS multilayer by continuous brush‐printing. Reproduced with permission.^[^ ^123^ ^]^ Copyright 2013, Elsevier B.V.

In terms of the advances of photodetectors with excellent detection ability mentioned above, OPDs exhibit an anticipative potential in flexible and stretchable applications, such as health monitoring, optical communication, and biomedical sensors and images, which is profiting from the significant properties of increasing sensitivity, tunable absorption characteristics, and mechanical flexibility.^[^ ^106^, ^107^ ^]^ As we know, the universally used substrate is glass, which is rigid and not befitting to be used as flexible substrates. Therefore, the progress has been made to find the replacement of the rigid glass. Thin and flexible plastic foils, such as PI (polyimide), PET (polyethyleneterphthalate), and poly(ethylene naphthalate) (PEN), which are widely used as the substrates in flexible electronics because of their high optical transparency in the visible region and good stability. Krebs et al. are the first to fabricate wearable OPDs that employ a traditional structure of PET/PEDOT:PSS/MEH‐PPV:PCBM/Al.^[^ ^108^ ^]^ Flexible PT‐OPDs based on plastic textiles were also demonstrated in past years.^[^ ^109^ ^]^ Therefore, polyimide (PI) substrates stand out due to their durability of high‐temperature processing. In 2018, Zhong et al. first fabricated PI‐based flexible OPDs with the ultrashort channel length, which showed excellent flexibility due to their unique structure design, as shown in **Figure** **14**a.^[^ ^110^ ^]^

Besides the substrates, it is essential to improve the mechanical flexibility of transparent conductive electrodes (TCEs) to construct flexible OPDs.^[^ ^111^ ^]^ As the most widely used electrode, ITO shows excellent optical transmittance and electrical conductivity in various optoelectronics, and enormous advances have been made based on it. However, it is not suitable for flexible devices because of its high brittleness. Therefore, it is urgent to develop flexible TCEs with comparable transparency, resistance, and roughness to ITO, by which the flexible devices can exhibit comparable performance to rigid counterparts. With the rapid development of flexible TCEs, there have been many alternatives in the replacement of ITO electrodes, such as silver nanowires (AgNWs), graphene, metal meshes, carbon nanotubes, high‐conductivity PEDOT:PSS, and hybrid composite electrodes.^[^ ^112^ ^]^ Though most researches of TCEs are based on organic solar cells (OSCs) and organic light‐emitting diodes (OLEDs), the proposed TCEs also apply to OPDs.

The most promising alternatives are the metallic nanostructures that possess high conductivity and malleability. Among them, AgNWs electrodes stand out because they demonstrate not only perfect electrical conductivity in flexible/stretchable state due to their high aspect ratio and percolation network structures, but also have the advantages of optical transparency and scalable solution processability.^[^ ^113^ ^]^ In 2019, Sun et al. presented a high property flexible TCE with AgNWs, which showed a low sheet resistance of around 10 Ω sq^−1^ and high transmittance of around 92%.^[^ ^114^ ^]^ Similarly, Zhang et al. reported a synergetic transparent electrode structure combining AgNWs with a moth‐eye nanopatterned ZnO layer, which was used for achieving broadband light trapping and alleviating the surface roughness (Figure 14b).^[^ ^115^ ^]^ Device efficiency of 11.02% was obtained for the device using PM6:IT‐4F as an absorber. Besides, metal meshes electrodes (especially Ag meshes) were also competitive alternatives in replacing conventional ITO electrodes, which showed not only great optical and electrical property but also high mechanical toughness and cost‐competitiveness. However, the corresponding fabrication process needed simplification to reduce the cost and increase the reproducibility.

Graphene is also considered as the promising flexible TCE in organic optoelectronics. It is a zero‐bandgap semi‐metal with ambipolar electrical characteristics which shows excellent mechanical properties with high thermal conductivity and commendable optical properties (up to 97.7% transmittance in the visible region). In terms of flexible TCE, there is a trade‐off between optical transmittance and electrical conductivity in single layer graphene due to its high resistance.^[^ ^116^ ^]^ In 2019, Wang et al. reported a flexible and efficient organic solar cell based on PET substrates with a three‐layer graphene TCE, in which the sheet resistance of graphene was increased to 260 Ω sq^−1^, along with a relatively low transmittance of 80% at the wavelength beyond 480 nm (Figure 14c).^[^ ^117^ ^]^ After optimizing, the flexible device showed comparable performance with devices based on ITO electrodes. Besides, there is another method in balancing the trade‐off between optical and electrical properties. Lee et al. fabricated a new TCE by inserting an ultrathin graphene film between PI layer and PEDOT:PSS layer (Figure 14d).^[^ ^118^ ^]^ This TCE showed a lower sheet resistance below 50 Ω sq^−1^ and good transparency of higher than 80%. Moreover, with the effect of interlayers, organic optoelectronics exhibited long‐term stability and larger efficiency.

Besides, conductive polymer, especially high conductive PEDOT:PSS is also an alternative to flexible TCEs due to its optoelectronic properties, tunable electrochemical performance, and great mechanical flexibility. Organic compounds, such as ethylene glycol (EG) and methanol, are commonly added to increase the crystallinity of PEDOT:PSS during the film formation, which further increase the electrical conductivity of PEDOT:PSS.^[^ ^119^, ^120^, ^121^ ^]^

To tackle the disadvantages of various flexible TCEs, hybrid electrodes are then widely studied based on two or more kinds of above mentioned flexible TCEs. By combining the flexibility of PEDOT:PSS and low resistivity of AgNWs, the hybrid electrodes showed a low sheet resistance of 13.96 Ω sq^−1^ and high optical transparency of 80.48%. Later, Dong et al. reported a typical thin and fully flexible hybrid electrode by integrating the AgNWs networks with monolayer graphene and polymer film, which exhibited a low enough sheet resistance of 8.06 Ω sq^−1^ and high transmittance of 88.3%.^[^ ^122^ ^]^ In 2013, Lee et al. fabricated highly transparent and flexible multilayer electrodes with PEDOT:PSS/AgNWs/PEDOT:PSS hybrid materials by continuous brush‐printing (Figure 14e).^[^ ^123^ ^]^ Similarly, Ricciardulli et al. synthesized a hybrid high‐transparency AgNWs and graphene‐based TCE with low‐surface roughness of 4.6 nm and low sheet resistance of 13.7 Ω sq^−1^, which was versatile for a wide variety of optoelectronics with high‐performance.^[^ ^124^ ^]^ Recently, Chen et al. developed a hybrid electrode that incorporating PEDOT:PSS/graphene composite film with thermal‐introduced wrinkles for OSCs. The fabricated flexible TCE showed an excellent transmittance of 88.5% at 550 nm and a decreased sheet resistance of 147.5 Ω sq^−1^ compared to pristine graphene.^[^ ^125^ ^]^ To sum up, the flexible TCEs show great potential and promising future in flexible optoelectronic devices. But the further investigation that aiming at optical, electrical, and mechanical properties are in priority to construct highly efficient OPDs.

Base on the improved performance of TCEs, many efforts have been made in scientific research and industry application to render OPDs more practical in wearable devices. And it is known that different light detections can be obtained by using different active materials with specific absorption characteristics, which can also obtain different applications. One of widely used applications is the spectrophotometer that is applied to environmental monitoring, imaging, and surveillance. Conventional spectrometers generally employ diffraction gratings or dichroic prisms in broadband photodetectors to realize spectral selective detection. The complexity and high cost of their fabrication hinder the further commercialization.^[^ ^2^ ^]^ Therefore, there are many kinds of explorations for suitable materials and device structures. Tang et al. demonstrated a miniaturized spectrophotometer by incorporating an array of narrowband cavity photodetectors based on the PBTTT:PCBM blends with a resonant optical cavity structure, as shown in Figure 11b.^[^ ^100^ ^]^ They got an impressive result that the device showed a comparable spectral resolution in moisture detection compared to a commercial spectrometer. The presented spectrophotometers demonstrated a feasible and low‐cost alternative for humidity monitoring and had a potential application in wearable electronics.

**Figure 15:**
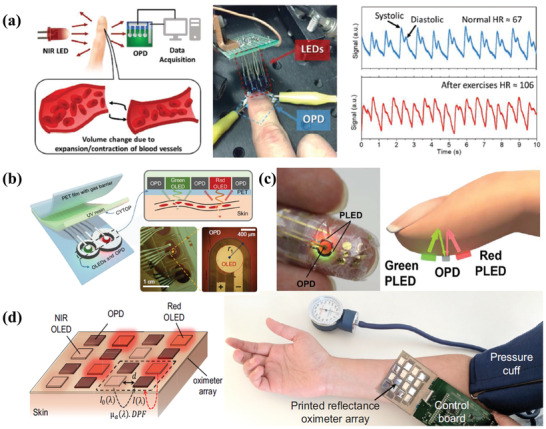
a) Basic working principle of PPG and the setups of the heart rate measurement using OPDs, along with the recorded pulse signals. Reproduced with permission.^[^ ^72^ ^]^ Copyright 2019, Wiley‐VCH. b) Schematic of the proposed OPO sensor with enlarged cross‐sectional view to depict device arrangement and light receiving process through the skin medium. Reproduced with permission.^[^ ^131^ ^]^ Copyright 2018, American Association for the Advancement of Science. c) The device structure and operation principle of the reflective pulse oximeter. Reproduced with permission.^[^ ^132^ ^]^ Copyright 2016, American Association for the Advancement of Science. d) The schematic configuration and photograph of the reflectance oximeter array. Reproduced with permission.^[^ ^134^ ^]^ Copyright 2018, The Authors. Published by PNAS.

Another common application is health monitoring and care, which catches our eyes due to the real‐time monitoring in the physiological conditions of individuals without the limitation in time and location. It is well‐known that most basic human features can be concluded through the blood flowing in the subcutaneous tissue, which uses NIR light primarily in wearable health‐monitoring devices due to its unique advantages.^[^ ^126^, ^127^ ^]^ Among these devices, photoplethysmography (PPG) stands out for health care and emergency diagnosis, which utilizes a low‐cost noninvasive optical technique to monitor health indicators accurately in heart rate and pulse pressure. For the PPG sensor, the key components are a photodetector, a light‐emitting diode, and a single collector.^[^ ^128^ ^]^ With the rapid development of NIR OPDs, the advance in PPG has been made. In 2020, Huang et al. reported a highly sensitive NIR organic photodiode based on an ultranarrow‐bandgap non‐fullerene acceptor, CO1‐4Cl, which showed a significant photoresponsivity of 0.5 A W^−1^ in the spectral region from 920 to 960 nm and a large detectivity of 10^12^ Jones.^[^ ^72^ ^]^ To test the practical application of this NIR OPD, they fabricated an integrated PPG, whose basic principle was shown in **Figure** **15**a. When NIR light emitted from OLEDs illuminated human tissues, it would be detected by an optical sensor with the changes in blood volume during every cardiac cycle. These changes could be converted into electrical signals, which would be collected to evaluate the heart rate. According to the recorded results, the typical systolic and diastolic peak in a PPG profile could be identified and the heart rate was determined to be 67 and 106 beats min^−1^ under these two different conditions.

To broaden the range of detection in analyzing health features, pulse oximetry is used for measuring pulse rate and arterial blood oxygenation with non‐invasive medical sensors. Traditional pulse oximeters employ optoelectronic sensors that consist of two inorganic light‐emitting diodes (IOLEDs) and a single inorganic photodiode to measure the health situation of human, nevertheless, the disadvantages of inorganic‐based pulse oximeters in high‐cost, rigidity, and complicated fabrication have limited the commercial application.^[^ ^129^ ^]^ Hence, all‐organic pulse oximetry was provided by Lochner et al., which based on the integration of green and red OLEDs and OPDs. When compared with the commercially available oximeters, the all‐organic optoelectronic oximeter sensor accurately measured pulse rate and oxygenation with a small error of 1%.^[^ ^130^ ^]^ Later, Lee et al. exhibited a reflective patch‐type pulse oximetry sensor with the design of flexible OLEDs and organic photodiodes, which could operate at a low electrical power of 24 µW on average and showed great promise for all‐day wearable health monitoring systems(Figure 15b).^[^ ^131^ ^]^ Similarly, Yokota et al. demonstrated a flexible and comfortable reflective pulse oximeter by integrating ultra‐flexible green and red OLEDs with an OPD, which was laminated to the skin using adhesive tape with a thickness of 6 µm.^[^ ^132^ ^]^ The operation principle and device structure could be seen in Figure 15c. Under working mode, the pulsating photoplethysmogram signal generated low noise and good repeatability. The fabricated device exhibited good stability in the air with this signal remaining constant after four days. Besides, the progress based on NIR sensitive OPDs had largely improved the performance of wearable pulse oximeters due to their long propagation distance in tissues and nontoxicity.^[^ ^133^ ^]^ In 2018, Khan et al. reported a reflectance oximeter array composed of four red and four NIR printed OLEDs and eight OPDs, as shown in Figure 15d.^[^ ^134^ ^]^ Employing organic optoelectronics and printing techniques, the reflectance oximeter was implemented with a small mean error of 1.1% compared with commercial transmission‐mode pulse oximeters.

Devices with stretchable property show unique application potential in wearable devices.^[^ ^135^ ^]^ In 2011, Lipomi et al. put P3HT:PC_60_BM layer onto PEDOT:PSS electrode with 200–500 µm prestretched polydimethylsiloxane (PDMS) substrate.^[^ ^136^ ^]^ The release of the tension created buckles in the active layer that accommodated subsequent cycles of strain. With further employed liquid metal back contact to improve mechanical stability, the device obtained a reversibly tensile strain of up to 27%. Jinno et al. fabricated a waterproof and stretchable device by encapsulating the device with acrylic elastomers.^[^ ^137^ ^]^ The device maintained 80% initial efficiency even after 20 cycles compression and 100 min exposure in water. In addition, fiber OPDs exhibit great potential as a typical kind of flexible and wearable optoelectronics due to their small volume and easier to large‐scale integration. Dong et al. fabricated a self‐powered UV photodetector based on fiber‐shaped through constructing a built‐in electric field between ZnO and polyvinylcarbazole (PVK), which was used to separate photogenerated charge carriers and resulted in a good responsivity of 9.96 mA W^−1^ under zero bias.^[^ ^138^, ^139^ ^]^ Notably, these photodetectors could stably operate under different bending state and show a good stretchable property, which could be woven into a web and bend at 90°.

## 7 OPDs in Integrated Devices

The outstanding features of OPDs make then promising in the realization of multifunctional devices. By rational designing the device structure, OPDs and other components are successfully integrated. One specific application of OPDs focusing on the combination with light‐emitting diodes (LED) is named organic upconverters (OUC). In 2005, Rossiter et al. fabricated a novel tactile sensor operating in both photo emitter and photodetector modes. Composing of a pliable foam surface over a substrate, this tactile sensor mounted a matrix of LEDs, which exhibited a two‐way detection. Later, relative applications are deeply researched. In 2011, Narasimhan et al. fabricated an integrated organic device with blue OLED and UV OPD with good performance under forward and reverse bias.^[^ ^140^ ^]^ By using a relatively new molecule 3,6‐dipyrenyl‐*N*‐hexylcarbazole (P2NHC) as the active layer, the integrated dual‐mode device possessed an efficient blue emitter with a low driving voltage of 2.7 V under forward bias and a prominent photodetector with a saturated photoresponsivity of 77 mA W^−1^ at 390 nm under the reverse bias.

**Figure 16:**
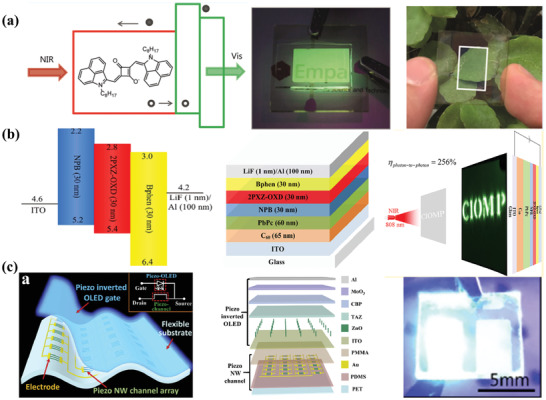
a) Transparent organic upconverter operated in the NIR mode. Reproduced with permission.^[^ ^142^ ^]^ Copyright 2018, American Chemical Society. b) Energy level diagram, device structure, and operating mode of NIR organic upconverter. Reproduced with permission.^[^ ^143^ ^]^ Copyright 2018, American Chemical Society. c) Schematics of the configuration of DPPT and photograph of the working device. Reproduced with permission.^[^ ^144^ ^]^ Copyright 2018, Elsevier Ltd.

Besides, NIR organic upconverters are essential to practical applications in biological imaging, communication, and optical sensors, which have made great progress in recent years.^[^ ^141^ ^]^ In 2018, Strassel et al. prepared a transparent OUC with an average visible transmittance of 65% and a peak transmittance of 80% at 620 nm, which combined a squaraine/fullerene OPD with a fluorescent Alq_3_‐based OLED.^[^ ^142^ ^]^ After optimization, the OPD showed a peak sensitivity at 980 nm and internal photon‐to‐current conversion efficiency of almost 100%. Based on this, the OUC could emit green light with a specific logo that was placed behind the upconverter in a dark room and irradiated with NIR light at 980 nm (**Figure** **16**a). Coincidentally, to solve the lower photo‐to‐photo conversion efficiency, Song et al. fabricated an all‐organic NIR‐to‐visible upconversion display with a photo multiplying NIR OPD with a high‐efficiency thermally activated delayed fluorescent OLED. The constructed device obtained a maximal photo‐to‐photo conversion efficiency of 256% that was much higher than 100%.^[^ ^143^ ^]^ Due to the suitable materials selection and device structure, the photodetector unit showed a photo multiplying effect and the upconverter exhibited an EQE of 10%. Under the illumination of NIR light with a shadow mask, a conformal image was obtained in the NIR upconverter (Figure 16b). As for other upconverters, Jiang et al. reported a high‐performance integrated dual piezo phototransistor (DPPT) recently.^[^ ^144^ ^]^ As the schematics of the configuration shown in Figure 16c, the OLED served as the top gate and horizontal ZnO nanowires array acted as a charge transport channel. Under stimulation, piezo‐phototronic effect was excited and further led to the enhancement of photoelectrical properties. The device showed a higher current on/off ratio of 10^6^ under mechanical deformation than that without deformation. Due to the easy integration of DPPT with photoelectrical technologies, this work provided a strategy for the development of integrated wearable devices.

To compensate for the disadvantages of integrated IPDs with the complicated fabrication process and high manufacturing expense, quantum dots organic blends, and organic‐inorganic hybrid materials are employed.^[^ ^145^ ^]^ In 2018, Xu et al. reported metasurface‐integrated OPDs with an enhanced photoresponsivity to achieve a relatively broadband improvement of the efficiency.^[^ ^146^ ^]^ The metasurface, which could prolong the light propagation path and increase the absorption, was fabricated on a silicon oxide‐coated silicon wafer using e‐beam lithography. Due to the internal electromagnetic resonant effect, a metasurface‐enhanced OPD was demonstrated with significantly increased absorption and photocurrent over a wavelength from 560 to 690 nm.

Though many advances have been made in improving the properties of OPDs and various applications are achieved in flexible, wearable, and integrated OPDs, an important issue still needs to be addressed is that the storing and operating stability of the OPDs.^[^ ^147^ ^]^ Hirsch et al. investigated the aging and reliability of the OPDs using PCDTBT:PC_60_BM blend films as absorber. They demonstrated OPDs with an ultra‐low dark current and a good detectivity under reverse bias, which also could operate continuously over 14 000 h in the long‐term aging tests.^[^ ^148^ ^]^ Besides, the reliability of unencapsulated OPDs was also monitored in the condition with both air and visible light. Kielar and co‐workers concluded that there was a dramatic reduction in the lifetime of OPDs and came out with a model to describe the nature of defects induced by the combination of air and light quantitatively.^[^ ^149^ ^]^ However, studies on the aging and reliability of OPDs are still insufficient, which is critical to the successful commercialization of this promising new technology. Considering OPDs are similar to OSCs in terms of device structures and materials, the aging and reliability researches on OSCs thus can provide guidance for OPDs.^[^ ^150^ ^]^ For example, Jinno et al. fabricated ultra‐flexible OSCs coated on both sides of elastomer with the structure of ITO/ZnO/Active layer/MoO_x_/Ag, which remained 80% of original PCE when compressed for 20 cycles and exposed to water for 100 min.^[^ ^137^ ^]^ In 2017, Kettle et al. developed a method that was used as the basis of lifetime testing models for OSC modules combining with data from ISOS consensus standards. Based on this, studies of the temperature‐, humidity‐, and light‐introduced degradation could be carried out during accelerated life testing against typical outdoor operation conditions.^[^ ^151^ ^]^ Most recently, Burlingame et al. studied the stability of single‐junction OSCs through accelerating the aging progress with cathode buffer layers. The fabricated devices could obtain an extrapolated intrinsic lifetime of more than 4.9 × 10^7^ and 1.7 × 10^4^ h when exposed to white‐illumination intensities of up to 37 and 20 Suns of ultraviolet illumination, respectively.^[^ ^152^ ^]^

## 8 Conclusion and Prospect

In summary, recent progress and different strategies to optimize the properties of OPDs have been summarized. Through the use of the PM effect, dual‐mode, suitable materials, and modified structures, the device performance has been significantly improved in detectivity, photosensitivity, EQE, and so on. Meanwhile, the spectral response has been extended from UV to infrared wavelength reigon, which also facilitates the practical application of OPDs. However, the preponderance of OPDs in the optoelectrical market is still limited as compared to that of inorganic conterparts, which is determined by the unsatisfactory electrical property of organic semiconductors and relatively low overall properties. By taking advantage of mechanical flexibility, spectral tunability, and ease of the device fabrication, OPDs are widely used in wearable optoelectrical devices, such as spectrophotometers, pulse oximeters, and biomedical imaging, which can be on a par with commercial optoelectronics. Therefore, it is anticipated that there will be attractive and practical applications in optoelectronic sensors and wearable products with the continuous enlargement in performance of OPDs in the future.

## Conflict of Interest

The authors declare no conflict of interest.
